# [^68^Ga]Ga-DOTA-FAPI-04 PET/MR in patients with acute myocardial infarction: potential role of predicting left ventricular remodeling

**DOI:** 10.1007/s00259-022-06015-0

**Published:** 2022-11-03

**Authors:** Min Zhang, Weiwei Quan, Tianqi Zhu, Shuo Feng, Xinyun Huang, Hongping Meng, Run Du, Zhengbin Zhu, Xuezheng Qu, Ping Li, Yuke Cui, Kuangyu Shi, Xiaoxiang Yan, Ruiyan Zhang, Biao Li

**Affiliations:** 1 Department of Nuclear Medicine, Ruijin Hospital, Shanghai Jiao Tong University School of Medicine, 197 Ruijin 2nd Road, Shanghai, 200025 China; 2 Collaboration Innovation Center for Molecular Imaging of Precision Medicine, Ruijin Center, Shanghai, China; 3 Department of Cardiovascular Medicine, Ruijin Hospital, Shanghai Jiao Tong University School of Medicine, 197 Ruijin 2nd Road, Shanghai, 200025 China; 4 Department of Informatics, Technical University of Munich, Munich, Germany; 5 Department of Nuclear Medicine, University of Bern, Bern, Switzerland

**Keywords:** Acute myocardial infarction, Myocardial fibrosis, Left ventricular remodeling, Fibroblast activation protein inhibitor, PET/MR

## Abstract

**Purpose:**

To assess predictive value of ^68^Ga-labeled fibroblast activation protein inhibitor-04 ([^68^Ga]Ga-DOTA-FAPI-04) PET/MR for late left ventricular (LV) remodeling in patients with ST-segment elevated myocardial infarction (STEMI).

**Methods:**

Twenty-six patients with STEMI were included in the study. [^68^Ga]Ga-DOTA-FAPI-04 PET/MR was performed at baseline and at average 12 months after STEMI. LV remodeling was defined as >10% increase in LV end-systolic volume (LVESV) from baseline to 12 months.

**Results:**

The LV remodeling group demonstrated higher [^68^Ga]Ga-DOTA-FAPI-04 uptake volume (UV) at baseline than the non-LV remodeling group (*p* < 0.001). [^68^Ga]Ga-DOTA-FAPI-04 UV at baseline was a significant predictor (OR = 1.048, *p* = 0.011) for LV remodeling at 12 months after STEMI. Compared to clinical information, MR imaging and cardiac function parameters at baseline, [^68^Ga]Ga-DOTA-FAPI-04 UV demonstrated better predictive ability (AUC = 0.938, *p* < 0.001) for late LV remodeling, with sensitivity of 100.0% and specificity of 81.3%.

**Conclusions:**

[^68^Ga]Ga-DOTA-FAPI-04 PET/MR is an effective tool to non-invasively quantify myocardial fibroblasts activation, and baseline [^68^Ga]Ga-DOTA-FAPI-04 UV may have potential predictive value for late LV remodeling.

**Supplementary Information:**

The online version contains supplementary material available at 10.1007/s00259-022-06015-0.

## Introduction

Heart failure after acute ST-segment elevated myocardial infarction (STEMI) causes complex and long-term structural cardiac changes known as left ventricular (LV) remodeling, and may be associated with poor prognosis. Myocardial fibrosis after acute myocardial infarction (AMI) plays a key role in LV remodeling [1]. Fibroblasts are the primary mediators of this response. Following AMI, activated fibroblasts migrate to the infarcted myocardium and produce large amounts of extracellular matrix proteins, promoting the repair and maintaining the structural integrity of the affected tissue. Although initial replacement of dead cardiomyocytes and fibrosis are essential to prevent ventricular wall rupture after ischemic injury, severe myocardial fibrosis increases ventricular stiffness and chamber dilatation, leading to late LV remodeling and cardiac dysfunction and heart failure.

Fibroblast activation protein (FAP) is a membrane-bound serine protease highly expressed in activated fibroblasts in the infarcted myocardium [2]. Positron emission computed tomography (PET) with Gallium 68-labeled FAP inhibitor (FAPI) targeting activated myocardial fibroblasts may provide a visual monitoring tool to help understand the pathogenesis of AMI. Recent studies have showed that FAPI uptake increased on the myocardium of patients with a history of coronary artery disease [3], pulmonary arterial hypertension [4], dilated cardiomyopathy [5], type II diabetes, other diseases associated with myocardial injury, and after chemotherapy or chest radiotherapy [6]; nonetheless, uptake was low in normal myocardium and myocardial scars [7]. Furthermore, a pioneering study in a rat model [8] and clinical studies in patients with AMI [7, 9–12] showed intense focal uptake of FAPI at the site of occlusion, and suggested a significant correlation between myocardial fibroblasts activation and myocardial injury in the early post-infarction period [10] and LV ejection fraction (LVEF) at 3~6 months follow-up [11, 12]. Therefore, SNMMI image of the year 2022 [12] showed FAPI PET as a new potential biomarker for predicting severity of cardiac remodeling after heart attack. However, there is still lack of data from a long-term study to reveal the predictive value of myocardial fibroblast activation measured by FAPI PET in the early phase of STEMI for late LV remodeling.

Our prospective study using hybrid PET/MR with ^68^Ga-labeled FAP inhibitor-04 ([^68^Ga]Ga-DOTA-FAPI-04) aimed to quantitatively assess the longitudinal changes in the intensity and extent of myocardial fibroblasts activation, and explore its predictive value for late LV remodeling at average 12 months after AMI.

## Materials and methods

### Subjects

**Fig. 1:**
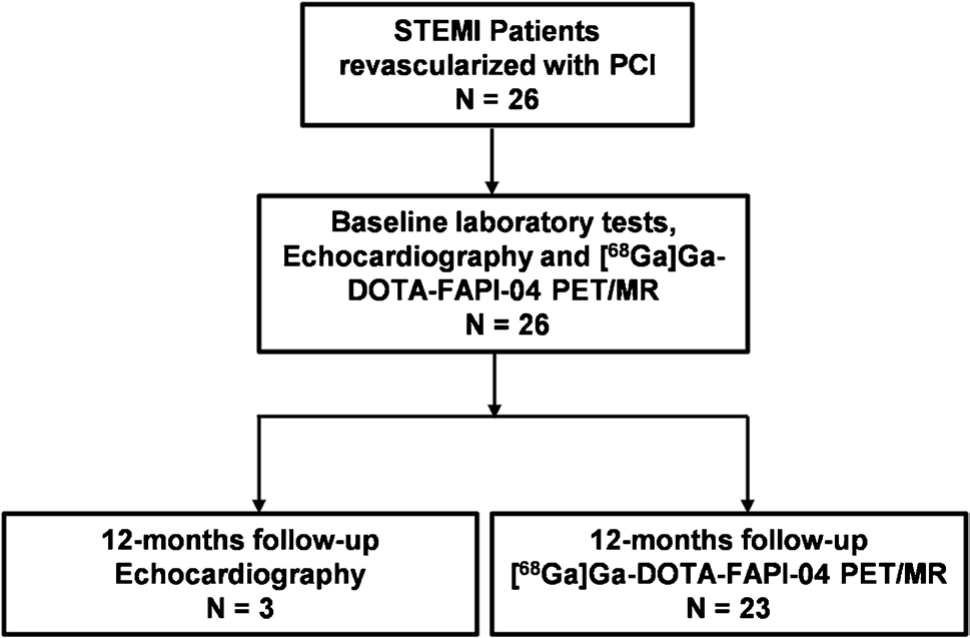
Study Flow Chart. STEMI, ST-segment elevated myocardial infarction

Twenty-six patients with STEMI were consecutively recruited from August 2020 to September 2021 and underwent primary percutaneous coronary intervention (PCI) within 12 h of symptom onset. All patients underwent baseline cardiac [^68^Ga]Ga-DOTA-FAPI-04 PET/MR scans at average 4.5 ± 1.5 days (3 ~ 8 days) after STEMI. Clinical symptom rating of the patients with AMI in this study was based on the Killip class [13]. Blood biomarkers tests, including serum peak N-terminal pro-brain natriuretic peptide (NT-pro-BNP_peak_), peak high-sensitivity C-reaction protein (hsCRP_peak_), low-density lipoprotein cholesterol (LDL-C), glycated hemoglobin (HbA1c), peak creatine kinase MB (CK-MB_peak_), and peak troponin I (TnI_peak_) were performed 24 h after AMI. Baseline echocardiography was performed at the day before discharge. Twenty-three of 26 patients underwent follow-up [^68^Ga]Ga-DOTA-FAPI-04 PET/MR scans at average 12 months (11.5 ± 1.4 months) after AMI. Other three patients refused 12-months follow-up [^68^Ga]Ga-DOTA-FAPI-04 PET/MR, but performed follow-up echocardiography at the same time. The study flow chart is shown in Fig. 1.

All procedures involving human participants were conducted in accordance with the ethical guidelines of the Declaration of Helsinki and national regulations. The study was approved by the Research Ethics Committee of Ruijin Hospital affiliated to Shanghai Jiao Tong University School of Medicine (2020CER152) and was registered at ClinicalTrials.gov (NCT04723953). All patients gave written informed consent.

### [^68^Ga]Ga-DOTA-FAPI-04 PET/MR image acquisition

All patients underwent [^68^Ga]Ga-DOTA-FAPI-04 PET on Biograph mMR system (Siemens, Erlangen, Germany), and no preparation was required. The FAPI precursor DOTA-FAPI-04 was purchased from CSBio Ltd. (Shanghai, China) and radiolabeled as previously described [14]. A 30-min PET scan was performed approximately 60 min after the intravenous injection of 2.2 ± 0.2 MBq/kg of [^68^Ga]Ga-DOTA-FAPI-04. Images were reconstructed using the point-spread function algorithm with 172 × 172 pixels, four iterations, 21 subsets, and a filter with a full width at half maximum of 2 mm.

Short- and long-axis T2-weighted short-tau inversion recovery (STIR), short-axis cine, and late gadolinium enhancement (LGE) images were acquired. STIR images (repetition time, 750 ms; echo time, 140 ms; slice thickness, 8.0 mm; acquisition matrix, 270×270; field of view, 256×256 mm; voxel size 1.1×1.1×8.0 mm) were used to show the anatomy of the heart. Short-axis cine images (repetition time, 51.60 ms; echo time, 1.51 ms; slice thickness, 8.0 mm; acquisition matrix, 256×192; field of view, 340×287 mm; voxel size 1.3×1.3×8.0 mm) were acquired to calculate cardiac function parameters. A phase-sensitive inversion recovery gradient-recalled echo sequence (repetition time, 930.40 ms; echo time, 1.55 ms; slice thickness, 8.0 mm; acquisition matrix, 256×164; field of view, 350×263 mm; voxel size, 1.4×1.4×8.0 mm) was performed 10 min after the administration of the contrast agent (0.04 mmol/kg; Magnevist, Bayer) to produce LGE images.

### PET/MR Image data analysis

PET and MR images were transferred to Syngo.via workstation (Siemens, Erlangen, Germany) for data registration, integration, and measurement and were reviewed by two experienced nuclear medicine physicians blinded to clinical and biological data of patients.

A volume of interest (VOI) was placed on the left ventricle to obtain the maximum standardized uptake value (SUV_max_) of [^68^Ga]Ga-DOTA-FAPI-04 on the infarcted myocardium. The uptake volume (UV) of enhanced [^68^Ga]Ga-DOTA-FAPI-04, defined as the extent of activated myocardial fibroblasts, was automatically calculated using a threshold above mean SUV (SUV_mean_) + 2 standard deviations in the reference tissue (left ventricular blood pool). The SUV_mean_ of the infarcted myocardium was obtained by calculating the average activity within the UV. The intensity of myocardial fibroblasts activation was quantified using the maximum and mean target-to-background ratio (TBR_max_ and TBR_mean_), which were equal to the SUV_max_ and SUV_mean_ of the infarcted myocardium divided by the SUV_max_ and SUV_mean_ in the reference tissue, respectively.

LGE images were analyzed using Segment version 3.1 (http://segment.heiberg.se) [15]. Endocardial and epicardial borders were traced manually, excluding papillary muscles. Infarct size was expressed as LGE volume and the percentage of LGE volume (LGE%) in the LV, and was automatically calculated using the expectation-maximization, weighted-intensity, a priori information (EWA) algorithm [16]. The extent of microvascular obstruction (MVO%), defined as the percentage of low signal area in the LV on LGE images, was also automatically calculated. Transmural infarction was defined as more than 75% LGE area in each involved myocardial segment. The segment count of transmural infarction on a 17-myocardial segment model (Segment version 3.1) as the extent of transmural infarction was calculated for each patient.

Left ventricular end-diastolic volume (LVEDV), end-systolic volume (LVESV) and LVEF was calculated from short-axis cine images using MR cardiac analysis software (Siemens, Erlangen, Germany). The change in cardiac function parameter was defined as the percentage of the difference between the follow-up and the baseline relative to the baseline. LV remodeling was defined as >10% increase in LVESV from baseline to 12 months after AMI [17, 18]. Additionally, there are two other reasons for choosing this definition. First, under this definition, LVEF at the time of 12-month follow-up significantly decreased in the LV remodeling group compared to the non-LV remodeling group (Supplemental Figure 1), which was consistent with the outcome of adverse LV remodeling. Second, LVESV has the advantage of combining data on both volumetric assessment and systolic function.

### Echocardiography

Echocardiography (GE vivid E9, GE Healthcare, USA) was performed by an experienced researcher in our hospital. LVEDV, LVESV and LVEF was calculated using the biplane Simpson method in two-dimensional apical four-chamber views. For the three patients without follow-up PET/MR, the change in cardiac function parameter was calculated using baseline and follow-up data measured by echocardiography.

### Statistical analysis

All statistical analyses were performed using SPSS version 20.0 (IBM Corp., Armonk, NY, USA), MedCalc version 20.027 (MedCalc Software Ltd, Ostend, Belgium) and GraphPad PRISM version 7.0 (GraphPad Software, La Jolla, CA, USA). Data were expressed as mean ± standard deviation or number (%). Correlations between [^68^Ga]Ga-DOTA-FAPI-04 PET and blood biomarkers as well as cardiac function parameters were analyzed using 2-tailed Pearson correlation analysis. Univariate logistic regression was used to analyze the predictability of clinical risk factors, cardiac function and imaging parameters at baseline on LV remodeling occurrence at one year after AMI. A linear regression analysis was performed for the relationship between [^68^Ga]Ga-DOTA-FAPI-04 uptake and LV remodeling using the changes in LVESV or LVEDV as continuous dependent variable. The incremental predictive ability of ^68^Ga-FAPI-04 UV to conventional parameters was assessed by Harrell’s C-statistics calculated from a Cox proportional hazards regression model and integrated discrimination improvement (IDI) index based on logistic model [19]. Positive values of IDI indicate that the combined model has better diagnostic ability than single parameter. Comparison of [^68^Ga]Ga-DOTA-FAPI-04 UV at baseline between patients in the LV remodeling and non-LV remodeling groups as well as comparison of the change in LV volumes between patients with [^68^Ga]Ga-DOTA-FAPI-04 UV < 134.8 cm^3^ and [^68^Ga]Ga-DOTA-FAPI-04 UV ≥ 134.80 cm^3^ was performed using independent samples t-test. Receiver operating characteristic (ROC) analysis was used to assess the predicting accuracy of baseline [^68^Ga]Ga-DOTA-FAPI-04 UV for LV remodeling. P-values smaller than 0.05 were considered statistically significant.

## Results

### Patients’ characteristics

Twenty-six patients including 25 men and 1 woman with average age 62.0 ± 8.4 year were enrolled in this study. A total of 20 of the 26 patients (76.9%) were rated Killip class I, while the other 6 (23.1%) patients were rated Killip class II. 65.4% (17/26) of the patients’ culprit vessels were left anterior descending coronary artery (LAD), 11.5% (3/26) were left circumflex coronary artery (LCX), while 23.1% (6/26) were right coronary artery (RCA). Clinical characteristics in 26 patients were summarized in Table 1, and detailed in Supplemental Table 1. Cardiac functions and imaging parameters at baseline and follow-up were showed in Supplemental Table 2-3.

**Table 1:** Clinical characteristics of patients at baseline

	Patients
Number	26
Demographic and risk factors	
Age (year)	62.0 ± 8.4
Male (*n*, %)	25 (96.2)
BMI (kg/m^2^)	25.5 ± 2.8
SBP (mmHg)	126.1 ± 19.8
DBP (mmHg)	77.7 ± 12.3
Medical history	
Obesity (*n*, %)	4 (15.4)
Diabetes (*n*, %)	6 (23.1)
Hypertension (*n*, %)	15 (57.7)
CAD (*n*, %)	7 (26.9)
Killip classification	
I (*n*, %)	20 (76.9)
II (*n*, %)	6 (23.1)
III-IV (*n*, %)	0 (0)
Culprit vessel	
LAD (*n*, %)	17 (65.4)
LCX (*n*, %)	3 (11.5)
RCA (*n*, %)	6 (23.1)
Clinical presentation	
NT-pro-BNP_peak_ (pg/ml)	1666.0 ± 2417.2
hsCRP_peak_ (mg/L)	49.0 ± 75.7
LDL-C (mmol/L)	2.9 ± 0.9
HbA1c (%)	6.2 ± 1.6
CK-MB_peak_ (ng/ml)	170.5 ± 161.6
TnI_peak_ (ng/ml)	93.3 ± 86.8
LVEDV (ml)	123.4 ± 29.7
LVESV (ml)	63.4 ± 28.2
LVEF (%)	49.9 ± 11.0

Data were expressed as mean ± standard deviation, or numbers (%). BMI, Body Mass Index; CAD, coronary heart disease; CK-MB, creatine kinase MB; DBP, diastolic blood pressure; hsCRP, hypersensitive c reaction protein; HbA1c, glycated hemoglobin; IS, infarct size; LAD, left anterior descending coronary artery; LCX, left circumflex coronary artery; LDL-C, low-density lipoprotein cholesterol; NT-pro-BNP, N-terminal pro-brain natriuretic peptide; RCA, right coronary artery; SBP, systolic blood pressure; TnI, troponin I; LVEDV, left ventricular end-diastolic volume; LVESV, left ventricular end-systolic volume; LVEF, left ventricular ejection fraction

### Longitudinal change of myocardial [^68^Ga]Ga-DOTA-FAPI-04 uptake and its correlation with blood biomarkers and cardiac functional parameters

As shown in Table 2, neither TBR_max_ nor TBR_mean_ at baseline was correlated with the level of acute inflammation or myocardial damage-related markers such as NT-pro-BNP_peak_, hsCRP_peak_, CK-MB_peak_, and TnI_peak_ as well as cardiac functional parameters at baseline. There was no correlation between [^68^Ga]Ga-DOTA-FAPI-04 UV and NT-pro-BNP_peak_, hsCRP_peak,_ CK-MB_peak_, and TnI_peak_ at baseline either.

**Table 2:** Correlation between myocardial [^68^Ga]Ga-DOTA-FAPI-04 uptake and blood biomarkers and CMR-derived cardiac functional parameters at baseline

*N* = 26	TBR_max_		TBR_mean_		UV	
	*r*	*p*	*r*	*p*	*r*	*p*
Blood biomarkers						
NT-pro-BNP_peak_	−0.108	0.598	−0.084	0.682	0.185	0.367
hsCRP_peak_	−0.153	0.464	−0.130	0.536	0.276	0.181
CK-MB_peak_	−0.054	0.794	−0.147	0.473	0.032	0.876
TnI_peak_	−0.242	0.235	−0.287	0.155	0.008	0.968
Cardiac functional parameters						
LVEDV	−0.094	0.647	−0.186	0.363	0.680	< 0.001
LVESV	−0.093	0.652	−0.174	0.396	0.720	< 0.001
LVEF	0.121	0.556	0.188	0.358	−0.681	< 0.001

NT-pro-BNP, N-terminal pro-brain natriuretic peptide; hsCRP, hypersensitive c reaction protein; CK-MB, creatine kinase mb; TnI, troponin I; LVEDV, left ventricular end-diastolic volume; LVESV, left ventricular end-systolic volume; LVEF, left ventricular ejection fraction; TBR, target-to-background ratio; UV, uptake volume

**Fig. 2:**
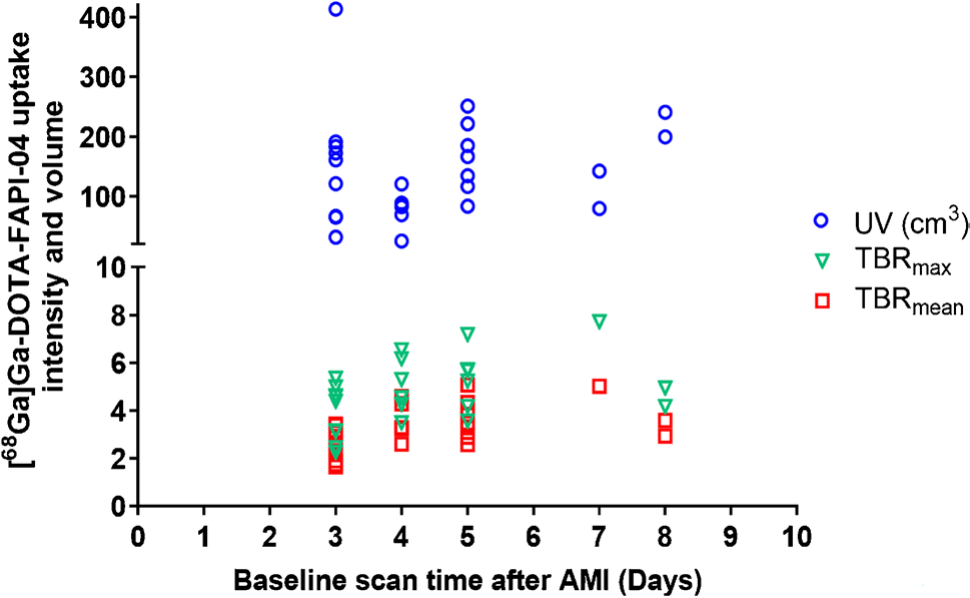
Myocardial [^68^Ga]Ga-DOTA-FAPI-04 uptake intensity and volume at baseline. TBR_max_ (green triangle) and TBR_mean_ (red square) as well as [^68^Ga]Ga-DOTA-FAPI-04 UV (blue circles) varied considerably between individuals. TBR: target-to-background ratio. UV: uptake volume

The volume and intensity of [^68^Ga]Ga-DOTA-FAPI-04 uptake at baseline varied considerably between individuals (Fig. 2). [^68^Ga]Ga-DOTA-FAPI-04 UV but not TBR_mean_ or TBR_max_ was significantly correlated with LVEDV (*r* = 0.680, *p* < 0.001), LVESV (*r* = 0.720, *p* < 0.001) and LVEF (*r* = −0.681, *p* < 0.001) at baseline (Table 2).

Both volume and intensity of [^68^Ga]Ga-DOTA-FAPI-04 uptake decreased from baseline to 12-month follow-up, but myocardial [^68^Ga]Ga-DOTA-FAPI-04 uptake could still be observed as long as approximately 12 months after AMI in all patients of our study. Correlation analysis demonstrated that [^68^Ga]Ga-DOTA-FAPI-04 UV but not TBR_mean_ or TBR_max_ at the time of 12-month follow-up was significantly associated with an increase in LVEDV (*r* = 0.445, *p* = 0.033) and LVESV (*r* = 0.456, *p* = 0.029) and a decrease in LVEF (*r* = −0.423, *p* = 0.044) during 12 months. Meanwhile, [^68^Ga]Ga-DOTA-FAPI-04 UV (*r* = −0.783, *p* < 0.001) and TBR_max_ (*r* = −0.484, *p* = 0.019) showed a negative correlation with LVEF at the time of 12-month follow-up (Supplemental Table 4).

**Fig. 3:**
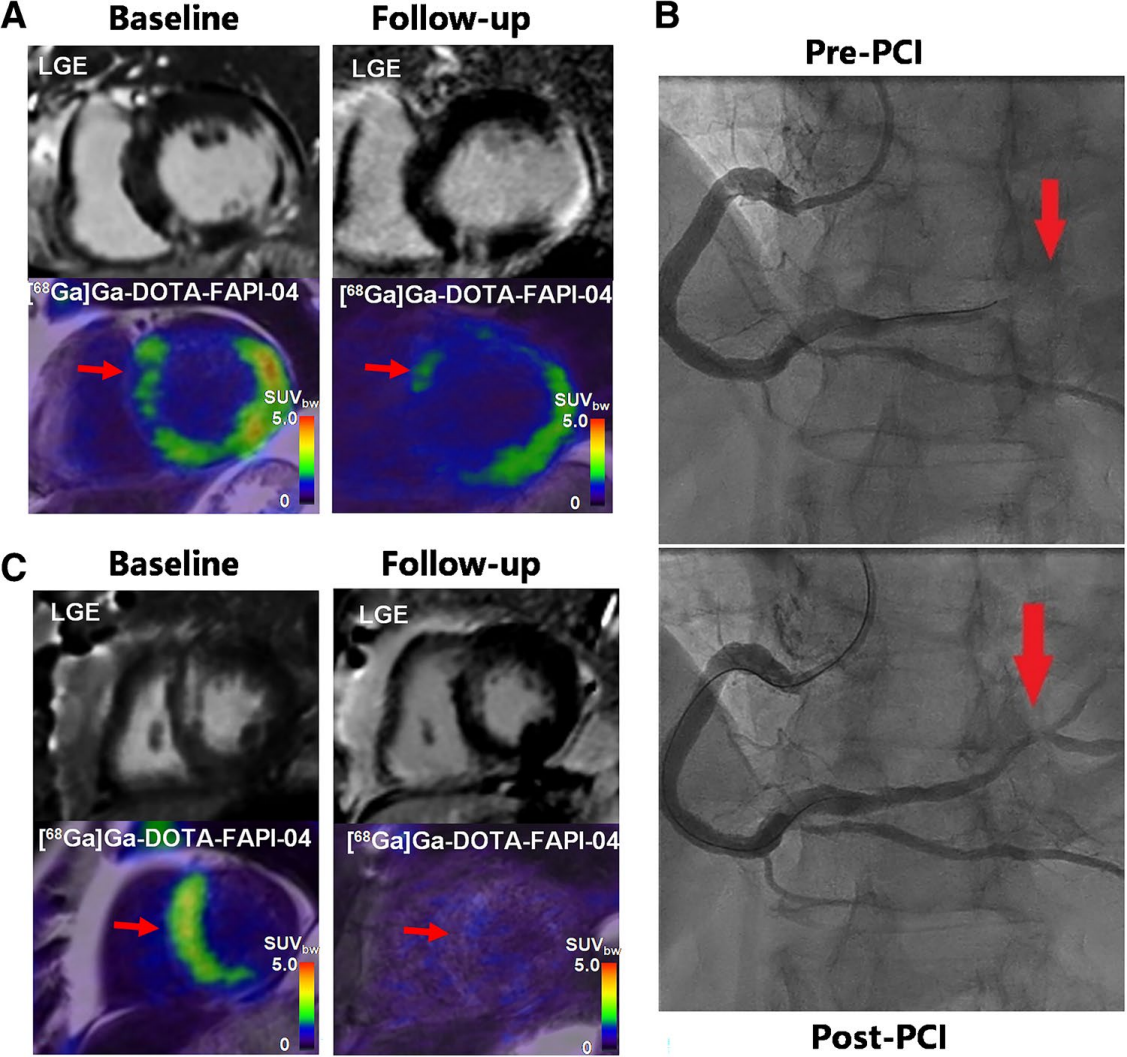
Representative [^68^Ga]Ga-DOTA-FAPI-04 PET/MR images in a patient with late LV remodeling and a patient with good cardiac functional outcomes. **A** In patient no. 6 (male, 66 years old, with RCA occlusion), large [^68^Ga]Ga-DOTA-FAPI-04 UV was observed at both baseline (251.3 cm^3^) and follow-up (229.3 cm^3^). More importantly, LVEDV and LVESV increased by 17.6% and 32.6%, respectively, and LVEF decreased by 28.6%, indicating adverse LV remodeling. Additionally, [^68^Ga]Ga-DOTA-FAPI-04 uptake was observed in the remote non-LGE area (red arrow). **B** Cardiac angiography showed an occlusion and a recanalization of a distal branch of RCA (red arrow) before and after PCI, suggesting microscopic myocardial infarction. **C** In patient no. 3 (male, 64 years old, with LAD occlusion), small [^68^Ga]Ga-DOTA-FAPI-04 UV (red arrow) at baseline (82.8 cm^3^) and follow-up (15.4 cm^3^) was observed. LVEDV and LVESV respectively decreased by 3.8% and 0.0% with follow-up 60% LVEF indicating good cardiac functional outcomes. LVEDV: left ventricular end-diastolic volume; LVESV: left ventricular end-systolic volume; LVEF: left ventricular ejection fraction; UV: uptake volume; LAD: left anterior descending coronary artery; RCA right coronary artery

Representative PET/MR images showed that a large extent of myocardial fibroblasts activation may be associated with increased LV volume and decreased LVEF in Patient No.6 (Fig. 3A). Additionally, [^68^Ga]Ga-DOTA-FAPI-04 uptake was observed at the remote non-LGE area in this patient. Cardiac angiography showed an occlusion of distal branch of RCA that dominated this area (Fig. 3B). It might be related to microscopic myocardial infarction due to the occlusion of distal small branch of culprit vessel, which is sensitively detected by [^68^Ga]Ga-DOTA-FAPI-04 PET but not by LGE MR imaging. In contrast, a small [^68^Ga]Ga-DOTA-FAPI-04 UV in patient no. 3 was associated with a good cardiac functional outcome (Fig. 3C).

### The relationship between baseline [^68^Ga]Ga-DOTA-FAPI-04 UV and late LV remodeling

**Fig. 4:**
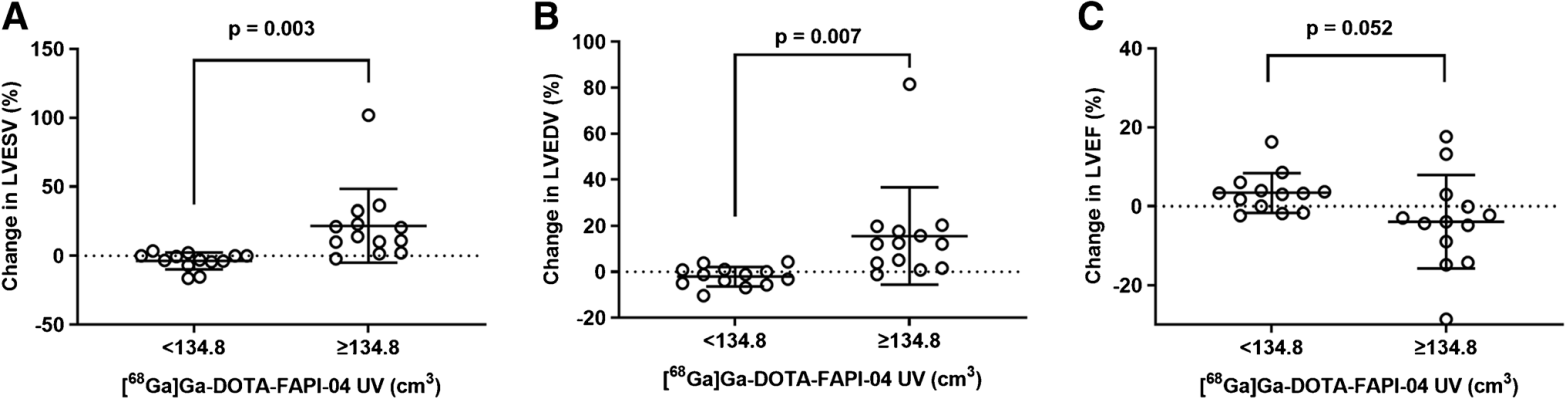
Comparison of the changes in cardiac function parameters between the group with [^68^Ga]Ga-DOTA-FAPI-04 UV < 134.8 cm^3^ and the group with [^68^Ga]Ga-DOTA-FAPI-04 UV ≥ 134.80 cm^3^. There was significant difference of the change in **A** LVESV (*p* = 0.003) and **B** LVEDV (*p* = 0.007) as well as a tendency in difference of (C) LVEF (*p* = 0.052) between the two groups. LVEDV: left ventricular end-diastolic volume; LVESV: left ventricular end-systolic volume; LVEF: left ventricular ejection fraction; UV: uptake volume

Based on the above observed association between longitudinal [^68^Ga]Ga-DOTA-FAPI-04 uptake and changes in cardiac function, we hypothesized that baseline [^68^Ga]Ga-DOTA-FAPI-04 UV may be associated with late LV remodeling. To verify our assumptions, we divided the patients into the group with [^68^Ga]Ga-DOTA-FAPI-04 UV < 134.8 cm^3^ (*n* = 13) and the group with [^68^Ga]Ga-DOTA-FAPI-04 UV ≥ 134.80 cm^3^ (n = 13) according to the median of [^68^Ga]Ga-DOTA-FAPI-04 UV at baseline. The changes in cardiac function parameters between the two groups were compared. As shown in Fig. 4, there was significant difference of the change in LVESV (*p* = 0.003) and LVEDV (*p* = 0.007) as well as a tendency in difference of LVEF (*p* = 0.052) between the two groups indicating a potential association between baseline [^68^Ga]Ga-DOTA-FAPI-04 UV and late increased LV volume and decreased LVEF.

**Fig. 5:**
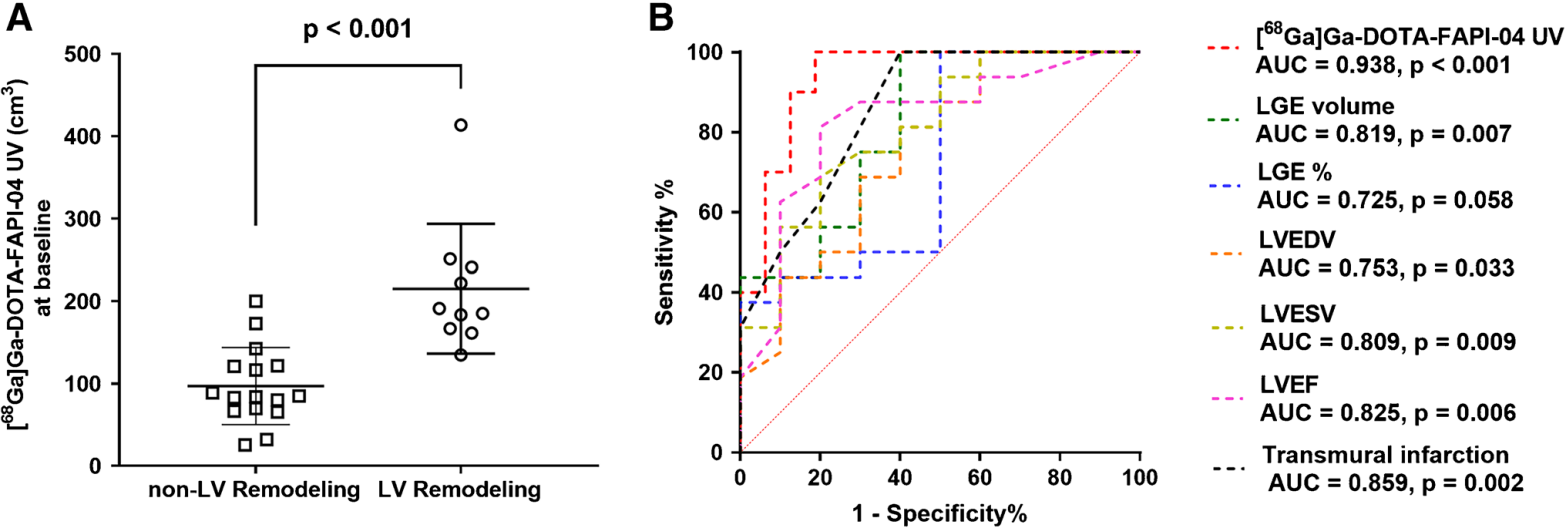
The relationship between baseline [^68^Ga]Ga-DOTA-FAPI-04 UV and late LV remodeling. **A** The LV remodeling group demonstrated higher [^68^Ga]Ga-DOTA-FAPI-04 UV at baseline than the non-LV remodeling group (*p* < 0.001). **B** Compared to LGE volume, LGE%, LVEDV, LVESV, LVEF, and the extent of transmural infarction, [^68^Ga]Ga-DOTA-FAPI-04 UV at baseline showed the highest AUC (0.938) for predicting the LV remodeling, with optimal sensitivity of 100.0% and specificity of 81.3%. UV: uptake volume; LV: left ventricular; LGE: late gadolinium enhancement; LVEDV: left ventricular end-diastolic volume; LVESV: left ventricular end-systolic volume; LVEF: left ventricular ejection fraction

Therefore, we further divided the patients into the LV remodeling (*N* = 10) and non-LV remodeling (*N* = 16) groups. We found that the LV remodeling group demonstrated higher baseline [^68^Ga]Ga-DOTA-FAPI-04 UV compared to the non-LV remodeling group (p < 0.001), as shown in Fig. 5A. Subsequently, we assessed the predictive ability of conventional clinical and CMR parameters as well as [^68^Ga]Ga-DOTA-FAPI-04 PET for late LV remodeling. Univariate logistic regression analysis showed that besides baseline cardiac function parameters, [^68^Ga]Ga-DOTA-FAPI-04 UV (OR = 1.048, *p* = 0.011), LGE volume (OR = 1.065, *p* = 0.021), LGE% (OR = 1.082, *p* = 0.035), and the extent of transmural infarction (OR = 2.320, *p* = 0.011) were significant predictors for LV remodeling at 12 months after STEMI (Table 3). Meanwhile, the relationship between [^68^Ga]Ga-DOTA-FAPI-04 uptake and LV remodeling when the latter is defined as a continuous variable for the changes in LVESV or LVEDV was performed. We found that [^68^Ga]Ga-DOTA-FAPI-04 UV was still a significant predictor (*B* = 0.116, *p* = 0.044) using the change in LVESV as a dependent variable, but it only showed a predictive trend without statistical significance (*B* = 0.080, *p* = 0.071) using the change in LVEDV as a dependent variable (Table 4). Additionally, combination of [^68^Ga]Ga-DOTA-FAPI-04 UV and conventional MR parameter such as LGE volume (IDI = 0.302, *p* = 0.002) and LGE% (IDI = 0.407, *p* < 0.001) significantly increased predictive accuracy compared to MR parameter alone (Table 5) suggesting the incremental value of [^68^Ga]Ga-DOTA-FAPI-04 UV for predicting late LV remodeling.

**Table 3:** Predictability of clinical risk factors, cardiac function and imaging parameters at baseline on late LV remodeling at 12 months after AMI

*N* = 26	Univariate logistic regression		
	OR	95% CI for OR	*p*
Clinical risk factors			
Sex	NA		1.000
Age (y)	1.011	0.917~1.114	0.830
LAD culprit vessel	0.682	0.131~3.546	0.649
Hypertension	2.333	0.439~12.398	0.320
Diabetes	0.750	0.110~5.109	0.769
Hyperlipidemia	1.667	0.092~30.060	0.729
Cardiac function parameters			
LVEF (%)	0.871	0.779~0.975	0.016
LVEDV (ml)	1.043	1.004~1.084	0.030
LVESV (ml)	1.057	1.008~1.108	0.021
Imaging parameters			
TBR_max_	0.950	0.700~1.289	0.743
TBR_mean_	0.899	0.581~1.390	0.632
UV (cm^3^)	1.048	1.011~1.087	0.011
LGE volume (ml)	1.065	1.010~1.124	0.021
LGE (%)	1.082	1.006~1.165	0.035
MVO (%)	2.354	0.753~7.364	0.141
Transmural infarction (*n*)	2.320	1.212~4.442	0.011

OR, odds ratio; CI, confidence interval; NA, not applicable; LAD, left anterior descending coronary artery; LVEDV, left ventricular end-diastolic volume; LVESV, left ventricular end-systolic volume; LVEF, left ventricular ejection fraction; TBR, target-to-background ratio; UV, uptake volume; LGE, late gadolinium enhancement; MVO, microvascular obstruction

**Table 4:** Predictability of baseline TBR_max_, TBR_mean_ and UV on the changes in LVESV or LVEDV from baseline to 12 months

*N* = 26	Change in LVESV			Change in LVEDV		
	*B*	95% CI for *B*	*p*	*B*	95% CI for *B*	*p*
TBR_max_	−2.945	−15.136~9.246	0.621	−0.984	−10.396~8.428	0.830
TBR_mean_	2.708	−13.012~18.428	0.724	0.553	−11.583~12.690	0.926
UV (cm^3^)	0.116	0.003~0.230	0.044	0.080	−0.007~0.168	0.071

LVEDV, left ventricular end-diastolic volume; LVESV, left ventricular end-systolic volume; TBR, target-to-background ratio; UV, uptake volume

**Table 5:** Improvement of accuracy of risk prediction using combination of conventional parameters and [^68^Ga]Ga-DOTA-FAPI-04 UV

	*C*-statistics	*p* value	IDI	*p* value
LVEF	0.825	NA	Ref	NA
LVEF+UV	0.938	0.15	0.476	<0.001
LVEDV	0.753	NA	Ref	NA
LVEDV+UV	0.938	0.05	0.587	<0.001
LVESV	0.813	NA	Ref	NA
LVESV+UV	0.938	0.127	0.528	<0.001
LGE%	0.725	NA	Ref	NA
LGE%+UV	0.938	0.049	0.407	<0.001
LGE volume	0.819	NA	Ref	NA
LGE volume+UV	0.944	0.125	0.302	0.002
MVO%	0.716	NA	Ref	NA
MVO%+UV	0.956	0.024	0.467	<0.001

NA, not applicable; Ref, reference; IDI, integrated discrimination improvement; LVEDV, left ventricular end-diastolic volume; LVESV, left ventricular end-systolic volume; LVEF, left ventricular ejection fraction; UV, uptake volume; LGE, late gadolinium enhancement; MVO, microvascular obstruction

Moreover, ROC analysis was also performed to compare the predictive performance of various parameters for late LV remodeling. Compared to baseline LGE volume (AUC = 0.819, *p* = 0.007), LGE% (AUC = 0.725, *p* = 0.058), LVEDV (AUC = 0.753, *p* = 0.033), LVESV (AUC = 0.809, *p* = 0.009), LVEF (AUC = 0.825, *p* = 0.006), and the extent of transmural infarction (AUC = 0.859, *p* = 0.002), [^68^Ga]Ga-DOTA-FAPI-04 UV at baseline showed the highest diagnostic performance (AUC = 0.938, *p* < 0.001) for predicting late LV remodeling, with optimal sensitivity of 100.0% and specificity of 81.3% (Fig. 5B).

## Discussion

Our initial longitudinal observational study demonstrates that [^68^Ga]Ga-DOTA-FAPI-04 PET/MR may be an effective tool to non-invasively quantify activated myocardial fibroblasts. Baseline [^68^Ga]Ga-DOTA-FAPI-04 UV may have a predictive value for late LV remodeling in patients with STEMI.

Longitudinal [^68^Ga]Ga-DOTA-FAPI-04 PET imaging allowed observing dynamic changes of activated myocardial fibroblasts after STEMI. Infarct healing has two phases: inflammatory (0–4 days), characterized by robust immune cell infiltration and tissue digestion, and reparative (lasting 10–14 days), manifested by myofibroblast conversion and proliferation and tissue repair [20]. Increased extracellular interstitial volume caused by edema in the inflammatory phase is rapidly demonstrated as increased signal on LGE images [21], while the activation of myocardial fibroblasts, which are generally originated from fibroblasts in response to TGF-ꞵ and other growth factors including platelet-derived growth factor along with the impact of neutrophils [22], is relatively delayed. Although activation of cardiac fibroblasts is driven by inflammatory factors, neither intensity nor volume of [^68^Ga]Ga-DOTA-FAPI-04 uptake at baseline was correlated with the level of acute inflammation or myocardial damage-related markers in the present study. Recent studies also showed that there was no correlation between FAPI uptake and the level of TGF-β1, TNF-α, IL-6, hsCRP [11], CK_peak_, and LDH_peak_ [10]. This may be due to the inconsistency of the peak time between these laboratory markers in the blood circulation and [^68^Ga]Ga-DOTA-FAPI-04 uptake in the myocardial tissue. This hypothesis needs to be further validated in future studies by dynamic assessment at different time points.

Different from [^68^Ga]Ga-DOTA-FAPI-04 uptake in the animal model of myocardial infarction declined to background levels within 2 weeks due to decreased number of activated myofibroblasts and increased apoptosis of myofibroblasts during the healing phase (14 days to several months after AMI), [^68^Ga]Ga-DOTA-FAPI-04 uptake of infarcted myocardium in the patients of our study could be observed as long as 12 months after AMI, suggesting that activated myocardial fibroblasts involved in human cardiac repair or LV remodeling exist much longer than that in the animal model. Furthermore, [^68^Ga]Ga-DOTA-FAPI-04 uptake that persisted at the 12-month follow-up may be correlated to the adverse LV remodeling that has already occurred in the past, and to a possible further LV remodeling in the future.

Although infarct size measured by LGE MR has been thought to be correlated with cardiac outcomes [23, 24] and the extent of transmural infarction strongly predicted LV remodeling after infarction [25], [^68^Ga]Ga-DOTA-FAPI-04 UV at baseline seems demonstrate better prediction of late LV remodeling compared to LGE volume, LGE %, and the extent of transmural infarction in our study. In fact, previous studies showed uncertain predictive power of myocardial LGE for late LV remodeling because of high individual variability, i.e., a portion of patients with large infarct size might not incur late LV remodeling on follow-up, while those with a limited infarct size might unexpectedly suffer late LV remodeling [23, 26]. Persistent and dysregulated inflammation response may be one of important participants in the development of late LV remodeling [26]. Since myocardial fibroblast is activated by inflammatory factors, [^68^Ga]Ga-DOTA-FAPI-04 UV may not only correlate with infarct size, but also indirectly reflect the extent of cardiac inflammatory response, which may be the reason why [^68^Ga]Ga-DOTA-FAPI-04 UV demonstrated better predictive power for late LV remodeling than conventional LGE MR. A recent study from Hannover medical school, Germany also suggested that there was a significant inverse correlation between FAPI volume and LVEF obtained at later follow-up [12].

However, it should be noted that although [^68^Ga]Ga-DOTA-FAPI-04 UV was a significant predictor of late LV remodeling, there was partial overlap in [^68^Ga]Ga-DOTA-FAPI-04 UV between the LV remodeling and non-LV remodeling groups. Previous studies have indicated that late LV remodeling occurrence may be also associated with other factors such as neurohormonal activations [27], humoral factors including cytokines and associated growth factors [28, 29], and chronic systemic inflammation due to metabolic causes including diabetes and hyperlipidemia [29] suggesting the complexity of mechanisms underlying the development of late LV remodeling after AMI.

Additionally, the lack of the role of TBR_max_ and TBR_mean_ in predicting LV remodeling may probably due to the high variability of [^68^Ga]Ga-DOTA-FAPI-04 uptake intensity within 2 weeks after AMI. As fibroblasts with increase of FAP expression level is gradually activated by inflammatory factors after PCI revascularization, myocardial [^68^Ga]Ga-DOTA-FAPI-04 uptake intensity seems to be dependent on time. In contrast, as fibroblasts at the border of infarct zone are the first to be activated [8], [^68^Ga]Ga-DOTA-FAPI-04 UV correlates mainly with infarct size, which may change less in the early period after AMI, and thus [^68^Ga]Ga-DOTA-FAPI-04 UV may have less time-dependent variability. Therefore, [^68^Ga]Ga-DOTA-FAPI-04 UV but not its intensity at baseline may be a promising predictive imaging indicator for late LV remodeling, which will likely make [^68^Ga]Ga-DOTA-FAPI-04 PET/MR an important workflow in the future for monitoring the efficacy of related drugs that act directing on the myocardial fibrosis process [30] or combat ongoing dysregulated inflammation [31].

The present study has the following limitations: (1) Only patients with relatively mild symptoms (Killip class I and II) who could tolerate long scan time of PET/MR were enrolled in the present study; therefore, data on patients with severe symptoms (Killip III and IV [13]), who are more likely to have serious LV remodeling and poor cardiac functional outcomes, were limited; (2) The inclusion of only one female patient in this study was a significant gender bias, and therefore a weakness in terms of generalization to the general population; (3) Because the limited sample size of this study was less suitable for multivariate logistic regression analysis, it is unclear whether clinical characteristics, cardiac function parameters, and MR imaging parameters at baseline would have an impact on the ability of [^68^Ga]Ga-DOTA-FAPI-04 UV to predict LV remodeling. Therefore, although [^68^Ga]Ga-DOTA-FAPI-04 UV have shown to be potentially valuable in predicting LV remodeling, strong conclusions still require larger samples to be obtained.

## Conclusions

Overall, [^68^Ga]Ga-DOTA-FAPI-04 PET/MR is an effective tool to non-invasively quantify myocardial fibroblasts activation, and baseline [^68^Ga]Ga-DOTA-FAPI-04 UV in the early stage of STEMI may have potential predictive value for late LV remodeling at 12 months follow-up.

## Supplementary information

ESM 1 (DOCX 73 kb)
