# Supplementary material for: [68Ga]Ga-DOTA-FAPI-04 PET/MR in patients with acute myocardial infarction: potential role of predicting left ventricular remodeling
Source: Eur J Nucl Med Mol Imaging. 2022 Nov 3;50(3):839–48. doi: 10.1007/s00259-022-06015-0 (PMC9852131; doi:10.1007/s00259-022-06015-0)

**Supplemental Table 1. Detailed clinical characteristics of patients at baseline**

| Serial No. | Gender | Age (y) | Culprit vessel | BMI | SBP (mmHg) | DBP (mmHg) | Obesity | Diabetes mellitus | Hypertension | CAD | Killip classification | LDL-C (mmol/L) | HbA1c(%) | CK-MB (ng/ml) | TnI (ng/ml) | hsCRP (mg/L) | NT-pro-BNP (pg/ml) |
| --- | --- | --- | --- | --- | --- | --- | --- | --- | --- | --- | --- | --- | --- | --- | --- | --- | --- |
| 1 | M | 60 | LCX | 26.0 | 94 | 59 | No | No | No | No | I | 1.2 | 5.3 | 148.8 | 261.3 | 5.8 | 664.5 |
| 2 | M | 55 | LAD | 27.5 | 140 | 87 | No | No | Yes | No | I | 1.6 | 6.5 | 18.4 | 82.2 | 271.3 | 3209.0 |
| 3 | M | 64 | LAD | 28.1 | 146 | 84 | Yes | Yes | Yes | No | I | 1.3 | 8.6 | 28.9 | 26.2 | 14.0 | 859.2 |
| 4 | M | 54 | LAD | 30.1 | 122 | 84 | Yes | No | Yes | No | I | 2.3 | 6.2 | 102.9 | 20.1 | 12.5 | 1070.0 |
| 5 | M | 64 | LCX | 25.3 | 102 | 76 | No | No | No | No | I | 3.5 | 6.2 | 469.1 | 142.5 | 19.3 | 822.8 |
| 6 | M | 66 | RCA | 27.1 | 134 | 82 | No | No | Yes | No | I | 4.3 | 6.0 | 512.2 | 104.0 | 91.3 | 1588.0 |
| 7 | M | 68 | LCX | 29.4 | 153 | 104 | Yes | No | Yes | Yes | I | 2.5 | 4.9 | 24.4 | 13.3 | 4.2 | 1755.0 |
| 8 | M | 64 | RCA | 26.4 | 97 | 57 | No | No | Yes | No | I | 2.5 | 5.3 | 390.0 | 138.1 | 30.4 | 2337.0 |
| 9 | M | 51 | LAD | 23.0 | 113 | 74 | No | No | Yes | No | I | 2.5 | 5.4 | 443.8 | 339.7 | 10.4 | 463.3 |
| 10 | M | 65 | RCA | 21.2 | 115 | 73 | No | No | No | Yes | I | 2.9 | 5.6 | 88.3 | 36.8 | / | 589.3 |
| 11 | F | 59 | RCA | 18.4 | 95 | 58 | No | Yes | No | No | II | 5.1 | 12.6 | 192.1 | 57.9 | 25.4 | 2826.0 |
| 12 | M | 62 | LAD | 22.0 | 116 | 76 | No | No | No | No | I | 2.8 | 5.4 | 4.8 | 4.5 | 45.3 | 348.3 |
| 13 | M | 56 | LAD | 22.5 | 119 | 79 | No | No | Yes | No | I | 4.3 | 5.2 | 431.5 | 171.1 | 13.9 | 848.3 |
| 14 | M | 60 | LAD | 22.9 | 131 | 87 | No | No | Yes | No | II | 3.2 | 5.6 | 2.5 | 104.7 | 8.7 | 793.4 |
| 15 | M | 52 | LAD | 23.2 | 131 | 87 | No | No | Yes | No | II | 2.3 | 5.3 | 40.4 | 9.4 | 5.5 | 1405.0 |
| 16 | M | 35 | LAD | 26.6 | 123 | 78 | No | No | No | No | I | 3.3 | 5.3 | 187.9 | 35.0 | 12.2 | 393.1 |
| 17 | M | 63 | LAD | 25.5 | 156 | 85 | No | Yes | Yes | No | I | 3.4 | 8.8 | 21.5 | 7.7 | 5.3 | 285.3 |
| 18 | M | 80 | LAD | 27.0 | 120 | 55 | No | No | No | Yes | I | 3.5 | 5.6 | 51.2 | 8.1 | 7.5 | 783.0 |
| 19 | M | 67 | LAD | 26.4 | 145 | 95 | No | Yes | Yes | Yes | II | 2.0 | 6.3 | 118.3 | 118.2 | 120.1 | 3152.0 |
| 20 | M | 66 | LAD | 26.1 | 143 | 82 | No | No | No | No | II | 2.7 | 5.0 | 37.8 | 101.2 | 281.01 | 12685.0 |
| 21 | M | 67 | RCA | 24.1 | 139 | 93 | No | No | No | No | II | 3.3 | 5.4 | 268.4 | 257.7 | 102.7 | 2073.0 |
| 22 | M | 62 | LAD | 28.4 | 106 | 64 | Yes | Yes | No | No | I | 3.1 | 5.7 | 289.3 | 136.5 | 36.6 | 1054.0 |
| 23 | M | 64 | LAD | 28.7 | 120 | 78 | No | No | No | Yes | I | 3.1 | 7.2 | 191.3 | 66.1 | 22.7 | 403.2 |
| 24 | M | 73 | LAD | 27.4 | 145 | 85 | No | Yes | Yes | Yes | I | 2.2 | 6.6 | 93.7 | 39.3 | 68.8 | 2057.0 |
| 25 | M | 65 | LAD | 24.5 | 107 | 67 | No | No | Yes | Yes | I | 3.3 | 6.2 | 230.7 | 50.7 | 6.0 | 542.0 |
| 26 | M | 69 | RCA | 25.4 | 166 | 70 | No | No | Yes | No | I | 2.9 | 5.6 | 46.0 | 93.5 | 5.1 | 309.4 |

BMI: Body Mass Index; CAD: coronary heart disease; CK-MB: creatine kinase mb; DBP: Diastolic blood pressure; hsCRP: hypersensitive c reaction protein; HbA1c: glycated hemoglobin; LAD: left anterior descending coronary artery; LCX: left circumflex coronary artery; LDL-C: low-density lipoprotein cholesterol; NT-pro-BNP: N-terminal pro-brain natriuretic peptide; RCA right coronary artery; SBP: Systolic blood pressure; TnI: troponin I

**Supplemental Table 2.** **Cardiac functions parameters at baseline and follow-up**

| Serial No. | Days after AMI | | LVEDV (ml) | | LVESV (ml) | | LVEF (%) | |
| --- | --- | --- | --- | --- | --- | --- | --- | --- |
|  | Baseline | Follow-up | Baseline | Follow-up | Baseline | Follow-up | Baseline | Follow-up |
| 1 | 5 | 357 | 133 | 124 | 57 | 59 | 55 | 54 |
| 2 | 8 | 414 | 134 | 155 | 66 | 80 | 51 | 49 |
| 3 | 4 | 370 | 79 | 76 | 31 | 31 | 60 | 60 |
| 4 | 4 | 366 | 120 | 114 | 68 | 57 | 43 | 50 |
| 5 | 7 | 413 | 120 | 121 | 65 | 66 | 46 | 45 |
| 6 | 5 | 332 | 193 | 227 | 138 | 183 | 28 | 20 |
| 7 | 3 | 367 | 173 | 194 | 114 | 130 | 34 | 33 |
| 8 | 5 | 398 | 137 | 144 | 73 | 81 | 46 | 44 |
| 9 | 3 | 380 | 163 | 161 | 98 | 108 | 34 | 35 |
| 10 | 3 | 353 | 127 | 132 | 70 | 77 | 45 | 41 |
| 11 | 4 | 357 | 93 | 92 | 46 | 44 | 50 | 53 |
| 12 | 5 | 358 | 128 | 124 | 58 | 54 | 55 | 57 |
| 13 | 3 | 346 | 116 | 117 | 45 | 46 | 61 | 60 |
| 14 | 5 | 352 | 118 | 143 | 65 | 79 | 45 | 45 |
| 15 | 7 | 343 | 92 | 91 | 34 | 33 | 62 | 64 |
| 16 | 4 | 323 | 103 | 104 | 43 | 43 | 58 | 59 |
| 17 | 4 | 320 | 79 | 79 | 31 | 30 | 61 | 63 |
| 18 | 3 | 306 | 120 | 122 | 47 | 46 | 53 | 60 |
| 19 | 5 | 303 | 172 | 206 | 110 | 135 | 36 | 34 |
| 20 | 3 | 430 | 92 | 167 | 52 | 104 | 44 | 38 |
| 21 | 3 | 293 | 145 | 130 | 72 | 61 | 51 | 53 |
| 22 | 5 | 314 | 104 | 117 | 41 | 56 | 61 | 52 |
| 23 | 3 | 293 | 135 | 141 | 71 | 69 | 47 | 51 |
| 24 | 8 | 283 | 141 | 158 | 93 | 95 | 34 | 40 |
| 25 | 4 | 270 | 103 | 107 | 34 | 34 | 66 | 68 |
| 26 | 3 | 289 | 88 | 83 | 26 | 26 | 70 | 69 |

AMI: Acute myocardial infarction; NT-pro-BNP: N-terminal pro-brain natriuretic peptide; LVEDV: left ventricular end-diastolic volume; LVESV: left ventricular end-systolic volume; LVEF: left ventricular ejection fraction;

**Supplemental Table 3.** **Imaging parameters** **at baseline and follow-up**

| Serial No. | TBR_max_ | | TBR_mean_ | | UV (cm^3^) | | LGE volume (ml) | | LGE (%) | | MVO (%) | | Transmural infarction segment count |
| --- | --- | --- | --- | --- | --- | --- | --- | --- | --- | --- | --- | --- | --- |
|  | Baseline | Follow-up | Baseline | Follow-up | Baseline | Follow-up | Baseline | Follow-up | Baseline | Follow-up | Baseline | Follow-up | Baseline |
| 1 | 3.6 | / | 2.6 | / | 116.5 | / | 39.5 | / | 27.0 | / | 0.7 | / | 4 |
| 2 | 4.9 | 3.6 | 3.6 | 2.7 | 241.2 | 88.8 | 115.1 | 29.8 | 58.3 | 22.9 | 10.7 | 0.0 | 7 |
| 3 | 3.5 | 1.9 | 4.3 | 1.6 | 82.8 | 15.4 | 16.8 | 5.9 | 14.5 | 6.2 | 0.0 | 0.0 | 2 |
| 4 | 5.3 | 2.5 | 3.3 | 1.9 | 88.7 | 9.9 | 56.0 | 3.8 | 37.2 | 3.8 | 0.4 | 0.0 | 5 |
| 5 | 7.7 | 2.8 | 5.0 | 1.8 | 142.5 | 42.0 | 46.8 | 12.8 | 37.8 | 12.0 | 0.9 | 0.0 | 4 |
| 6 | 5.2 | 2.5 | 2.9 | 1.8 | 251.3 | 229.3 | 59.8 | 25.2 | 28.1 | 13.7 | 1.3 | 0.2 | 4 |
| 7 | 5.3 | 4.4 | 3.4 | 2.0 | 413.7 | 311.2 | 44.0 | 23.6 | 20.0 | 14.9 | 0.1 | 0.0 | 2 |
| 8 | 4.2 | 3.1 | 3.5 | 2.2 | 134.8 | 85.3 | 26.5 | 29.3 | 21.8 | 24.5 | 0.1 | 0.1 | 6 |
| 9 | 4.6 | 3.9 | 2.7 | 2.1 | 183.7 | 96.4 | 67.9 | 71.8 | 47.4 | 50.0 | 2.0 | 2.4 | 7 |
| 10 | 4.4 | 3.7 | 3.4 | 2.5 | 191.2 | 106.7 | 24.9 | 25.1 | 23.3 | 23.0 | 0.0 | 0.0 | 5 |
| 11 | 4.3 | 3.0 | 2.6 | 1.7 | 69.4 | 34.4 | 10.3 | 7.7 | 12.0 | 7.6 | 0.1 | 0.0 | 1 |
| 12 | 5.7 | 1.7 | 4.3 | 1.4 | 83.7 | 16.7 | 5.9 | 2.6 | 4.0 | 1.9 | 0.0 | 0.0 | 0 |
| 13 | 4.6 | 3.9 | 3.1 | 2.5 | 121.5 | 95.4 | 46.9 | 35.2 | 34.0 | 22.3 | 1.5 | 0.1 | 3 |
| 14 | 5.7 | 5.0 | 3.3 | 2.3 | 185.2 | 180.3 | 59.3 | 39.5 | 44.4 | 29.7 | 1.0 | 0.4 | 6 |
| 15 | 17.8 | 1.9 | 13.7 | 1.7 | 79.9 | 14.0 | 23.9 | 18.6 | 20.7 | 19.4 | 0.2 | 0.3 | 2 |
| 16 | 6.2 | 2.7 | 4.6 | 2.0 | 120.8 | 43.5 | 39.3 | 17.7 | 34.2 | 21.4 | 0.4 | 0.1 | 5 |
| 17 | 4.5 | 2.4 | 3.2 | 1.9 | 25.3 | 5.5 | 6.0 | 4.0 | 5.4 | 4.2 | 0.0 | 0.0 | 0 |
| 18 | 5.0 | / | 3.1 | / | 172.9 | / | 34.2 | / | 30.0 | / | 0.1 | / | 3 |
| 19 | 7.2 | 3.5 | 5.1 | 2.0 | 221.8 | 147.6 | 74.0 | 37.0 | 50.4 | 29.0 | 1.2 | 0.1 | 7 |
| 20 | 3.2 | 3.2 | 2.3 | 2.1 | 161.4 | 128.4 | 90.9 | 45.9 | 51.3 | 31.4 | 5.3 | 0.1 | 6 |
| 21 | 3.1 | / | 1.9 | / | 65.3 |  | 43.0 | / | 31.4 | / | 2.3 | / | 5 |
| 22 | 5.7 | 3.3 | 3.8 | 2.4 | 166.9 | 93.4 | 35.9 | 31.2 | 27.3 | 25.1 | 0.7 | 0.1 | 3 |
| 23 | 2.5 | 2.0 | 1.7 | 1.8 | 66.5 | 19.7 | 31.1 | 22.9 | 28.4 | 23.7 | 0.2 | 0.1 | 2 |
| 24 | 4.2 | 4.7 | 2.9 | 2.5 | 200.0 | 168.9 | 57.5 | 37.7 | 41.6 | 28.9 | 0.4 | 0.1 | 4 |
| 25 | 6.5 | 3.2 | 3.3 | 2.3 | 84.9 | 36.0 | 14.0 | 11.3 | 14.6 | 15.3 | 0.1 | 0.1 | 0 |
| 26 | 2.3 | 1.5 | 1.7 | 1.5 | 31.9 | 5.6 | 9.2 | 6.9 | 11.0 | 8.4 | 0.0 | 0.0 | 0 |

LGE: late gadolinium enhancement; MVO: microvascular obstruction; TBR: target-to-background ratio; UV: uptake volume; ECV: extracellular volume

**Supplemental Table 4. Correlation between [^68^Ga]Ga-DOTA-FAPI-04 uptake at 12-month and the change in LVEDV, LVESV and LVEF as well as LVEF at 12-month follow-up**

|  | Change in LVEDV | |  | Change in LVESV | |  | Change in  LVEF | |  | LVEF at 12-month follow-up | |
| --- | --- | --- | --- | --- | --- | --- | --- | --- | --- | --- | --- |
|  | r | p |  | r | p |  | r | p |  | r | p |
| TBR_max_ | 0.319 | 0.138 |  | 0.276 | 0.202 |  | 0.004 | 0.987 |  | -0.484 | 0.019 |
| TBR_mean_ | 0.252 | 0.246 |  | 0.262 | 0.227 |  | -0.074 | 0.738 |  | -0.228 | 0.295 |
| UV | 0.445 | 0.033 |  | 0.456 | 0.029 |  | -0.423 | 0.044 |  | -0.783 | <0.001 |

LVEDV: left ventricular end-diastolic volume; LVESV: left ventricular end-systolic volume; LVEF: left ventricular ejection fraction; TBR: target-to-background ratio; UV: uptake volume

**Supplemental Figure 1. Comparison of the change in LVESV, LVEDV and LVEF between the LV remodeling and non-LV remodeling groups.** Both (A) LVESV (p < 0.001) and (B) LVEDV (p = 0.003) more significantly increased from baseline to 12-months follow-up, and (C) LVEF more significantly decreased (p < 0.001) in the LV remodeling group compared with the non-LV remodeling group. LV: left ventricular; EDV: end-diastolic volume; ESV: end-systolic volume; EF: ejection fraction


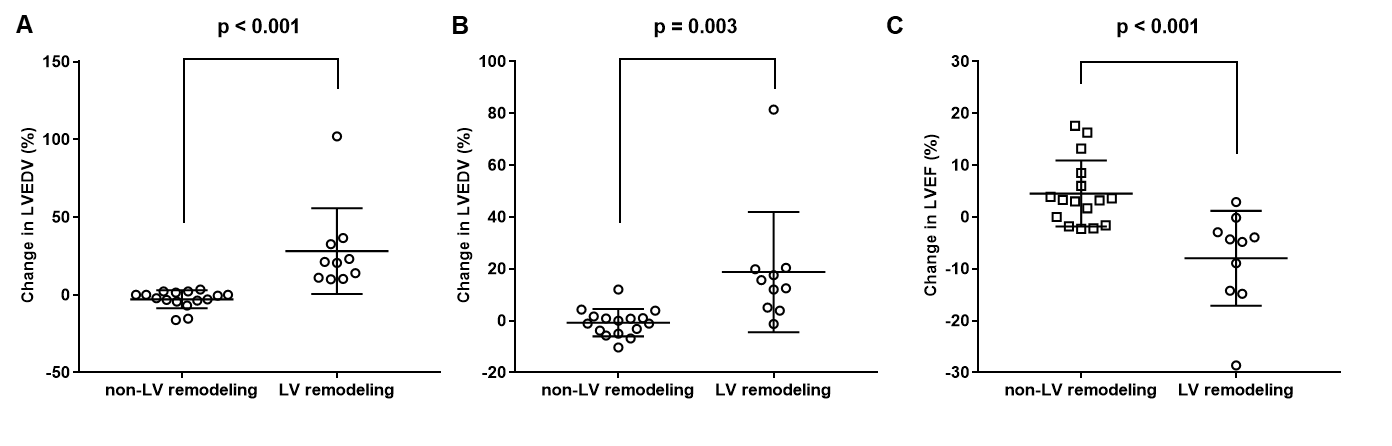

Supplement: Supplementary file 1 — (DOCX 73 kb) [file 259_2022_6015_MOESM1_ESM.docx]
